# Spanish Adaptation and Validation of the Transplant Effects Questionnaire (TxEQ-Spanish) in Liver Transplant Recipients and Its Relationship to Posttraumatic Growth and Quality of Life

**DOI:** 10.3389/fpsyt.2018.00148

**Published:** 2018-04-18

**Authors:** María Á. Pérez-San-Gregorio, Agustín Martín-Rodríguez, Milagrosa Sánchez-Martín, Mercedes Borda-Mas, María L. Avargues-Navarro, Miguel Á. Gómez-Bravo, Rupert Conrad

**Affiliations:** ^1^Department of Personality, Assessment, and Psychological Treatment, University of Seville, Seville, Spain; ^2^Department of Psychology, Universidad Loyola Andalucía, Seville, Spain; ^3^Hepatic-Biliary-Pancreatic Surgery and Liver Transplant Unit, University Hospital Virgen del Rocío of Seville, Seville, Spain; ^4^Department of Psychosomatic Medicine and Psychotherapy, University of Bonn, Bonn, Germany

**Keywords:** transplant effects questionnaire, TxEQ-Spanish, posttraumatic growth, quality of life, liver transplantation

## Abstract

The valid assessment of the impact of transplantation on psychological well-being is highly relevant to optimize treatment. However, to date there is no standardized instrument available in Spain. The Transplant Effects Questionnaire (TxEQ) evaluates the specific problems associated with organ transplantation, such as worry about transplant, guilt regarding the donor, disclosure of having undergone transplantation, adherence to medical treatment and responsibility toward the donor, family, friends, or medical staff. Against this backdrop the English original version of the TxEQ was translated into Spanish and validated in a sample of 240 liver transplant recipients. Participants also filled in the Posttraumatic Growth Inventory (PTGI), and the 12-Item Short Form Health Survey (SF-12v.2). Confirmatory factor analysis of the TxEQ-Spanish revealed a five-factor structure equivalent to the English original version, and satisfactory internal consistency (Cronbach's alpha: worry α = 0.82, guilt α = 0.77, disclosure α = 0.91, adherence α = 0.82, responsibility α = 0.83). Results showed that better mental quality of life was associated with higher adherence and disclosure, as well as less worry and guilt. Higher posttraumatic growth was significantly associated with worry, guilt, and responsibility. Interestingly, the most powerful predictor of posttraumatic growth was worry. Analysis of variance showed an interaction effect of PTG and mental quality of life on adherence, with medium PTG being associated with significantly stronger adherence in participants with better mental quality of life. In conclusion our study could successfully adapt and validate the Spanish version of the TxEQ in a large sample of liver transplant recipients. Our findings show a complex relationship between emotional reactions to transplantation, mental quality of life, and posttraumatic growth, which give further insight into inner processes supporting psychological well-being and adherence after liver transplantation.

## Introduction

Transplantation has a great impact on the patients' physical and psychological well-being. Standardized psychometric instruments such as the Transplant Effects Questionnaire (TxEQ) are important to be able to assess and compare these effects and may help to optimize treatment. The English original version of this questionnaire was developed and tested with kidney transplant recipients [1]. Later, it was translated to other languages. The German version (TxEQ-D) was validated in a group of heart, lung, liver, and kidney transplant recipients [2], the Dutch version (TxEQ-NL) in a group of liver transplant recipients [3], and the Polish version in a group of heart transplant recipients [4]. The factor structure of the above mentioned versions is similar to the original version consisting of the five subscales worry, guilt, disclosure, adherence, and responsibility. In the German version the five-factor structure was confirmed in Confirmatory Factor Analysis (CFA) by a scree plot [2] and in the Dutch version the Root Means Square Error of Approximation (RMSEA = 0.063) as well as the Akaike Information Criterion (AIC = 19578) confirmed good model fit [3]. For the Polish version no indices of model fit were presented [4]. Regarding reliability Cronbach's alpha varied between 0.71–0.79 in the German version [2], 0.66–0.79 in the Dutch version [3], and 0.61–0.72 in the Polish version [4] compared to 0.72–0.86 in the English original version [1].

Despite the growing importance of transplantation medicine in Spain [5] there is no Spanish translation of the TxEQ. The TxEQ has successfully been used to assess the impact of different forms of transplantation (living vs. deceased donor) [6, 7] and different organ types (heart, lung, liver, kidney) on quality of life. Thus, in a sample of 370 solid organ transplant recipients, 18.2% of liver recipients worried about the transplant compared to 14.4% of kidney recipients, 3.9% in lung recipients, and 2.4% of heart recipients [8]. Another study revealed that recipients after living donation tend to experience more guilt toward the donor compared to transplant recipients of deceased donors [7].

There is growing evidence that recipients' quality of life is closely connected to their ability to adapt to the new situation after a traumatic experience [9]. This ability has been described by Tedeschi and Calhoun [10, 11] as posttraumatic growth and several studies showed its relevance as a protective factor for psychological well-being after transplantation [12–15]. According to Tedeschi and Calhoun the experience of growth is often motivated by the experience of personal distress and worries and the inner need to find a new intra-psychic balance. The importance of worry as predictor of posttraumatic growth has been confirmed in cancer patients [16, 17]. A successful adaptation of the transplant recipient may result in a modification of coping strategies [18], a shift in priorities, and a change in family ties [19]. However, the complex relationship between specific emotional reactions to transplantation as measured by the TxEQ, posttraumatic growth and quality of life is not fully understood.

Against this backdrop our study aimed at an adaptation of the English TxEQ to Spanish and a validation of the Spanish version (TxEQ-Spanish) in a sample of liver transplant recipients. We had the following hypotheses. First, the TxEQ-Spanish has a five-factor structure similar to the original version with the subscales worry, guilt, disclosure, adherence, and responsibility. Second, posttraumatic growth is related to higher scores on worry, guilt, disclosure, adherence, and responsibility. Third, mental quality of life is related to lower scores on worry and guilt and higher scores on disclosure, adherence, and responsibility. Finally, in accordance with the theory of Tedeschi and Calhoun [10, 11] and previous studies in cancer patients [16, 17] we assumed worry to be the most powerful predictor among TxEQ scales of posttraumatic growth after transplantation.

## Materials and methods

### Participants and procedure

This research was approved by the Ethics Committee of the Virgen del Rocío University Hospital of Seville. All patients gave their informed consent for participation. A group of 240 liver transplant recipients was selected (185 men and 55 women), with a mean age of 60.21 ± 9.30 years. 79.2% had a stable partnership and 61.7, 22.5, and 15.8% had a low, medium, and high education, respectively. The average time that had elapsed since the transplant was 87.77 ± 66.19 months. The etiology of the liver disease was as follows: alcoholic cirrhosis (32.1%), hepatocellular carcinoma (27.9%), hepatitis C-related cirrhosis (17.1%), hepatitis B-related cirrhosis (5%), and others (17.9%). The liver received by all patients was from a donor who had died from the following causes: cerebrovascular accidents (59.9%), cranioencephalic traumas (27%), and others (13.1%). For the specific patient selection procedure and inclusion criteria see Pérez-San-Gregorio et al. [9].

### Instruments

#### Transplant effects questionnaire (TxEQ; [1])

The English original version consists of 23 items scored on a 5-point Likert scale ranging from “strongly agree” to “strongly disagree.” It contains five subscales that assess worry about the transplant (six items, e.g., “I am worried about damaging my transplant”), guilt regarding the donor (five items, e.g., “I feel guilty about having taken advantage of the donor”), disclosure (three items; e.g., “I avoid telling other people that I have a transplant”), adherence (five items, e.g., “Sometimes I do not take my anti-rejection medicines”), and responsibility (four items, e.g., “I think that I have responsibility to the transplant team to do well”). The score of each subscale is calculated by dividing the sum score by the number of items. Higher scores show a higher degree of the dimension concerned.

The factor structure was similar in all versions derived from this questionnaire, Table 1 presents subscales' internal consistency as measured by Cronbach's alpha.

**Table 1:** Cronbach's alpha for the TxEQ-Spanish and the English, German, Dutch, and Polish versions.

	**[1] (English)**	**[2] (German)**	**[3] (Dutch)**	**[4] (Polish)**	**TxEQ-Spanish**
Worry	0.81	0.73	0.68	0.61	0.82
Guilt	0.76	0.74	0.66	0.63	0.77
Disclosure	0.86	0.71	0.79	0.72	0.91
Adherence	0.79	0.79	0.78	0.61	0.82
Responsibility	0.72	0.73	0.66	0.63	0.83

#### Posttraumatic growth inventory (PTGI; [10])

This questionnaire consists of 21 items scored on a 6-point Likert scale (0 to 5) ranging from “no change” to “a very great degree of change,” thereby evaluating the perception of personal benefits in survivors of traumatic events. Test interpretation provides a total score of posttraumatic growth and the following five subdimensions: relating to others, new possibilities, personal strength, spiritual change, and appreciation of life. We used the Spanish version by Weiss and Berger [20]. Three equal-sized groups with different levels of posttraumatic growth (low, medium, high) were formed. A higher score showed more posttraumatic growth. Cronbach's alpha was 0.94 for the sum scale and 0.74 to 0.88 for the subscales.

#### 12-item short form health survey (SF-12v.2; [21, 22])

This instrument is made up of 12 items scored on either 3 or 5-point Likert-scales. It evaluates the following eight dimensions of health-related quality of life covering the previous 4 weeks: physical functioning, role-physical, bodily pain, general health, vitality, social functioning, role-emotional, and mental health. The sum score of the two components Physical Component Summary (PCS) and Mental Component Summary (MCS) was calculated by the Quality Metric Health Outcomes^TM^ Scoring Software 5.0. The sum score can range from 0 (worst state of health) to 100 (best state of health). Based on the liver transplant recipients' scores on the MCS, two groups of the same size with better or worse mental quality of life were formed. Cronbach's alpha was 0.92 for the PCS and 0.88 for the MCS [21].

### Translation and statistical analysis

Data were analyzed and graphics were produced using the software programs, Mplus v.7 [23] and SPSS 22 (IBM Corporation, Armonk, NY, United States) for Windows PC.

#### Translation of the transplant effects questionnaire (TxEQ) into spanish

The translation of the English original version of the TxEQ into Spanish strictly followed the guidelines for the process of cross-cultural adaptation of self-report measures by Beaton et al. [24] as well as the guidelines to quality control by Hambleton and Zenisky [25]. After requesting permission of the original authors the questionnaire was translated into Spanish by two psychology professors with advanced levels of English (stage I). After completion of both translations, translated items were compared and checked. Non-conformities were discussed until a consensus was reached and a final version was drafted (stage II). This version was translated back into English by two professional translators (stage III). On the basis of these translations and all previous reports a final version was produced by translators as well as other research team members (stage IV). This Spanish version was pilot tested for comprehensibility in a small group of transplant recipients (*n* = 10) (stage V). All participants confirmed the comprehensibility of the TxEQ-Spanish.

#### Statistical analysis

To validate the Spanish version of the TxEQ and to analyse the relationship between the TxEQ and quality of life as well as posttraumatic growth the following statistical analyses were applied.

A Confirmatory Factor Analysis (CFA) was performed to replicate the five-factor structure of the English original version of the TxEQ. In the first place, it was confirmed that the data matrix was adequate for factor analysis by measuring sample adequacy with the Kaiser-Meyer-Olkin Test (≥0.08) and the Bartlett test of sphericity (*p* ≤ 0.0001). To determine the best estimation method, the assumption of multivariate normality of the data was tested, checking to see whether the Mardia test [26] showed a standardized value over 5 [27]. For the fit indexes and model evaluation, the adequacy of the factorial solution was analyzed in several different ways: (a) indications of model fit: we took into account whether the Comparative Fit Index (CFI) and Non-Normed Fit Index (NNFI) or the equivalent Tucker and Lewis Index (TLI) values were near to or over 0.90, whether the Root Mean Square Error of Approximation (RMSEA) was less than 0.08 and whether the Test of Approximate Fit of RMSEA was non-significant [28, 29], and (b) significance of the parameters.

To analyze the internal consistency of the questionnaire, Cronbach's alpha coefficients were calculated for all dimensions, considering an internal consistency of at least 0.70 as adequate [30].

Pearson's chi-squared test was used to compare socio-demographic and clinical variables (gender, marital status, education, and etiology of the disease) in the patient subgroups. For the quantitative variables (age and time elapsed since transplantation), a one-way ANOVA was calculated. Before analysis of the relationship between TxEQ subscales, quality of life (SF12) and posttraumatic growth (PTGI) we checked data of the different scales for normality distribution by the Kolmogorov-Smirnov test, however scales were not normally distributed. The Levene-test for the equality of variances was not significant so that data showed homoscedasticity. According to statistical literature [31–33] ANOVA and Pearson's correlation produce reliable results under these circumstances given a large sample size of *n* = 240. A 3x2 factorial ANOVA was performed to evaluate the influence of posttraumatic growth level (low, medium, high) and MCS (worse, better) on transplantation effects. Pearson's correlation was used to analyze associations between TxEQ-Spanish dimensions, posttraumatic growth, and mental quality of life. Cohen's *d* (for quantitative variables) and Cohen's *w* (for qualitative variables) were computed as a measure of effect size. Effect sizes were interpreted as follows: for Cohen's *d* < 0.20=null effect size; ≥0.20<0.50=small; ≥0.50<0.80=moderate; ≥0.80=large. And for Cohen's *w* < 0.10=null effect size; ≥0.10<0.30=small; ≥0.30<0.50=moderate; ≥0.50=large [34].

Finally, we performed a stepwise multiple linear regression analysis to predict posttraumatic growth of liver transplant recipients (criterion or dependent variable) by means of seven predictor variables (worry, guilt, disclosure, adherence, responsibility, MCS, and PCS). Statistical requirements for the implementation of linear regression analysis (linearity, independence of residuals, homoscedasticity, no-multicollinearity) were fulfilled.

## Results

### Spanish adaptation and validation of the transplant effects questionnaire (TxEQ)

On an exploratory level, we confirmed that the matrix was suitable for factoring. Kaiser-Meyer-Olkin's measurement of sample adequacy was 0.80, which is considered satisfactory [35] and the Bartlett's sphericity test was not significant (*p* ≤ 0.0001).

On a descriptive level, participant responses did not show any missing values or outliers. The mean scores for all 23 items varied from 1.63 ± 0.93 to 4.66 ± 0.90, with skewness (−3.11, 1.78) and kurtosis (−1.48, 9.19) which deviated from the range of −1 and 1 required to consider distribution of the items normal [36]. There was no normal distribution of data (Mardia's coefficient = 36.68; value higher than 5.00; 27). Consequently, Robust Maximum Likelihood was employed for CFA calculation. Correlations between the TxEQ-Spanish subscales, posttraumatic growth and mental quality of life are presented in Table 2.

**Table 2:** Correlations between the TxEQ-Spanish subscales, posttraumatic growth, and mental quality of life.

	**Posttraumatic growth *r* (*p*)**	**Mental quality of life *r* (*p*)**	**Worry *r* (*p*)**	**Guilt^a^*r* (*p*)**	**Disclosure *r* (*p*)**	**Adherence *r* (*p*)**
Worry	0.37 (<0.001)	−0.22 (0.001)				
Guilt^a^	0.13 (0.046)	−0.13 (0.036)	0.37 (<0.001)			
Disclosure	0.02 (0.741)	0.19 (0.002)	−0.21 (0.001)	−0.43 (<0.001)		
Adherence	0.08 (0.238)	0.16 (0.014)	−0.09 (0.164)	−0.28 (<0.001)	0.44 (<0.001)	
Responsibility	0.25 (<0.001)	0.01 (0.876)	0.33 (<0.001)	0.14 (0.030)	−0.06 (0.319)	0.10 (0.120)

a *TxEQ [1] item 8 (“I do not have any feeling of guilt toward the donor”) was not included in the factor computation*.

**Figure 1:**
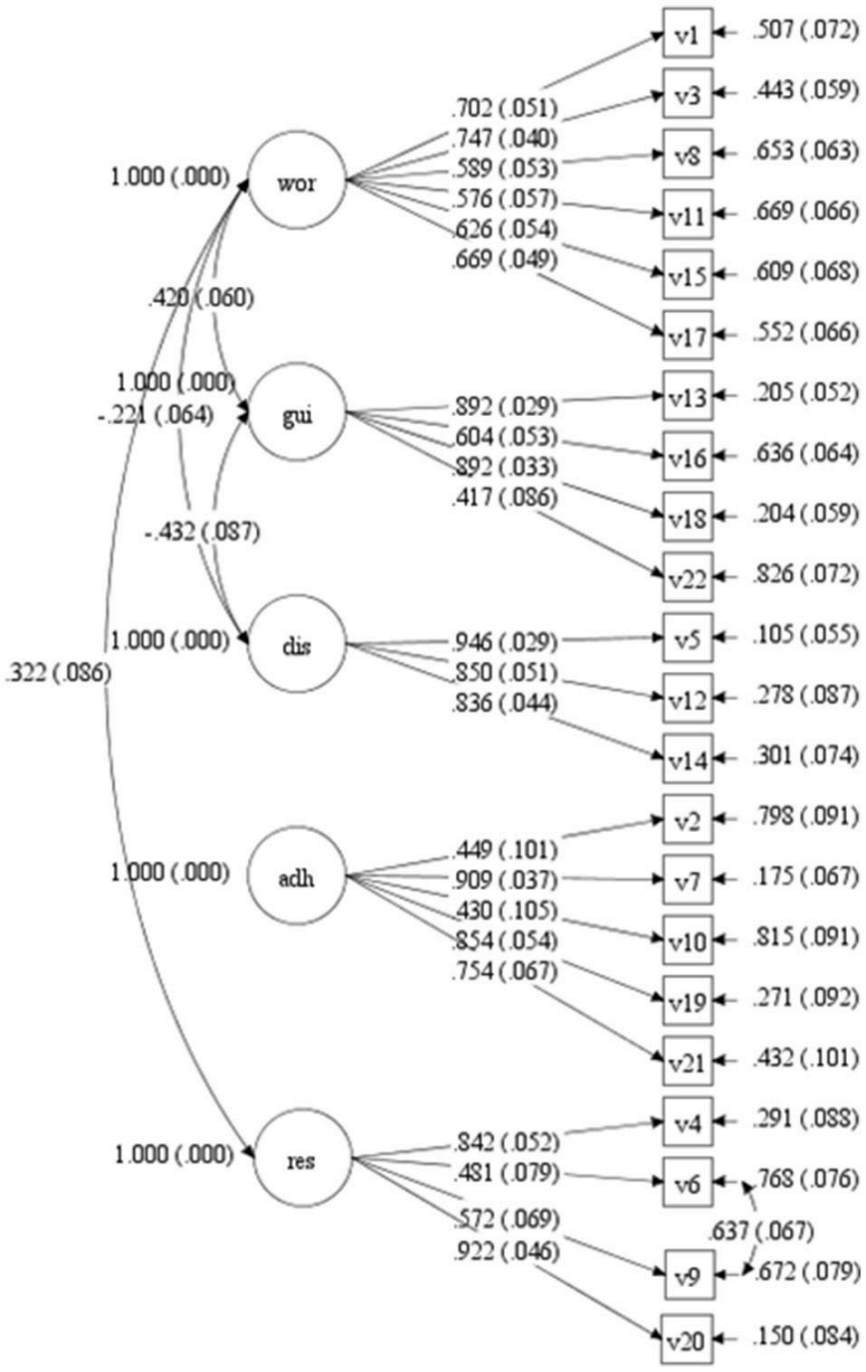
CFA diagram. Standardized factor loadings with standard error of the items and residuals and co-variance between dimensions.

In the next step of analysis the original five-factor structure was tested. Certain adjustments had to be made for satisfactory fit and significance of all the model parameters. Specifically, three adjustments were made to the original structure: First, a new parameter was added to the model, the residual covariance of items 6 and 9, both indicators of the same responsibility dimension (Figure 1). Second, significant correlations were allowed between the following dimensions: worry and guilt, worry and disclosure, worry and responsibility, and guilt and disclosure. There were no other significant inter-correlations. Third, item 8 (“I do not have any feeling of guilt toward the donor”) was eliminated, because it showed a saturation of < 0.2 and was negatively correlated to the corresponding dimension (guilt). The final version of the scale is shown in Appendix 1 in Supplementary Material. Moreover, in Appendix 2 in Supplementary Material we present a table comparing the TxEQ-Spanish and the English original version on an item to item basis.

After having made these adjustments, the fit indices were satisfactory: CFI = 0.90 and TLI = 0.89; RMSEA = 0.063 (H_0_: RMSEA < 0.05; *p* = 0.013; 90% confidence interval = 0.053–0.072). Figure 1 shows the diagram of the resulting model in which the standardized factor weights are given together with standard error and residuals for each item, as well as the covariance between dimensions and errors in items 6 and 9. All the estimated parameters were statistically significant (*p* < 0.01), the values were as follows: worry 0.58 to 0.75 (*M* = 0.65, *SD* = 0.05), guilt 0.42 to 0.89 (*M* = 0.70, *SD* = 0.05), disclosure 0.84 to 0.95 (*M* = 0.88, *SD* = 0.04), adherence 0.43 to 0.91 (*M* = 0.68, *SD* = 0.07), and responsibility 0.48 to 0.92 (*M* = 0.70, *SD* = 0.06). The proportion of variance explained by the predictor variables varied from 0.17 to 0.85. In the final model a correlation of *r* = 0.42 (*p* < 0.001) was observed between the factors worry and guilt, *r* = −0.22 (*p* = 0.001) between worry and disclosure, *r* = 0.32 (*p* = 0.001) between worry and responsibility, and *r* = −0.43 (*p* < 0.001) between guilt and disclosure. The correlation between errors on items 6 and 9 was *r* = 0.64 (*p* < 0.001).

Cronbach's alpha for internal consistency as a measure of reliability was satisfactory for all five subscales: worry 0.82, guilt 0.77, disclosure 0.91, adherence 0.82, and responsibility 0.83 (Table 1).

### Posttraumatic growth and mental quality of life: influence on the effects of the transplant

Based on patients' total score on the PTGI, three equal-sized subgroups were created: low score (*n* = 80 patients, 33.3% of the sample, 0 to 59 points), medium score (*n* = 80 patients, 33.3% of the sample, 60 to 77 points), and high score (*n* = 80 patients, 33.3% of the sample, 78 to 105 points). There were no significant differences between subgroups in age (*p* = 0.506), gender (*p* = 0.639, *w* = 0.06, null effect size), marital status (*p* = 0.720, *w* = 0.05, null effect size), education (*p* = 0.187, *w* = 0.16, small effect size), time elapsed since transplantation (*p* = 0.227), or etiology of the liver disease (*p* = 0.082, *w* = 0.24, small effect size). In a further step of analysis, another two subgroups of equal size were formed based on the SF-12 MCS: lower score or worse mental health (*n* = 120, 50% of the sample, ≤52.87 points) and higher score or better mental health (*n* = 120, 50% of the sample, >52.87 points). There were no significant differences between both subgroups regarding age (*p* = 0.105), gender (*p* = 0.091, *w* = −0.11, small effect size), marital status (*p* = 0.026, *w* = −0.14, small effect size), education (*p* = 0.075, *w* = 0.15, small effect size), time elapsed since transplantation (*p* = 0.926), or etiology of the liver disease (*p* = 0.442, *w* = 0.12, small effect size).

**Figure 2:**
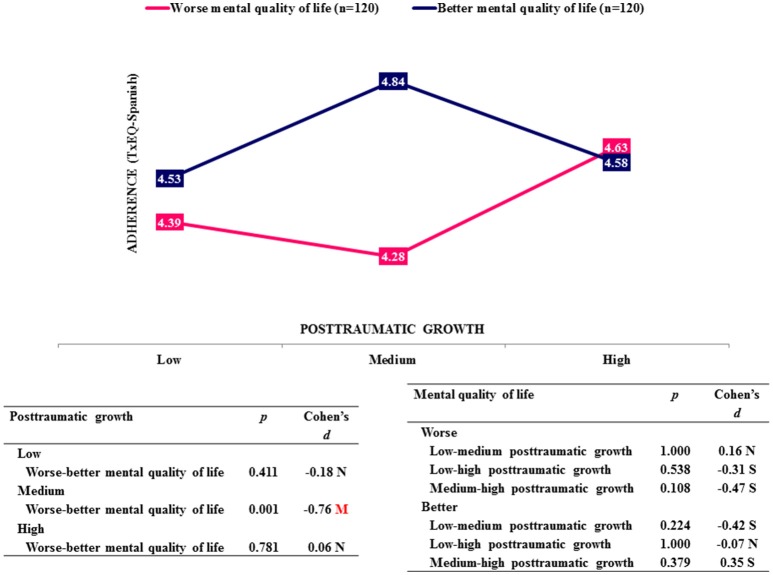
Simple effects on adherence. N, Null effect size; S, Small effect size; M, Medium effect size.

In the analysis of variance only the dimension adherence showed an interaction effect between the two factors posttraumatic growth and mental quality of life (*p* = 0.032) (Table 3). Figure 2 demonstrates this relationship. There was only a significant difference in adherence (*p* = 0.001, *d* = −0.76) between patients with worse and better mental quality of life if they showed medium posttraumatic growth; better quality of life was associated with stronger adherence of medium effect size. This difference disappeared when posttraumatic growth was low (*p* = 0.411, *d* = −0.18, null effect size) or high (*p* = 0.781, *d* = 0.06, null effect size). Moreover, as shown in Table 2, adherence showed a significantly positive correlation with mental quality of life (*r* = 0.16, *p* = 0.014) and disclosure (*r* = 0.44, *p* < 0.001), and a negative correlation with guilt (*r* = −0.28, *p* < 0.001).

**Table 3:** Influence of posttraumatic growth and mental quality of life on the TxEQ-Spanish subscales (3x2 factorial ANOVA).

	**Main effects**		**Interaction effects**
	**Posttraumatic growth *F*_(2, 234)_ (*p*)**	**Mental quality of life *F*_(1, 234)_ (*p*)**	***F*_(2, 234)_ (*p*)**
Worry	16.68 (<0.001)	14.45 (<0.001)	1.23 (0.295)
Guilt^a^	1.64 (0.195)	5.99 (0.015)	0.30 (0.739)
Disclosure	0.17 (0.845)	4.38 (0.037)	0.71 (0.493)
Adherence	0.76 (0.468)	5.11 (0.025)	3.49 (0.032)
Responsibility	6.41 (0.002)	0.00 (0.946)	0.55 (0.576)

a *TxEQ [1] item 8 (“I do not have any feeling of guilt toward the donor”) was not included in the factor computation*.

**Figure 3:**
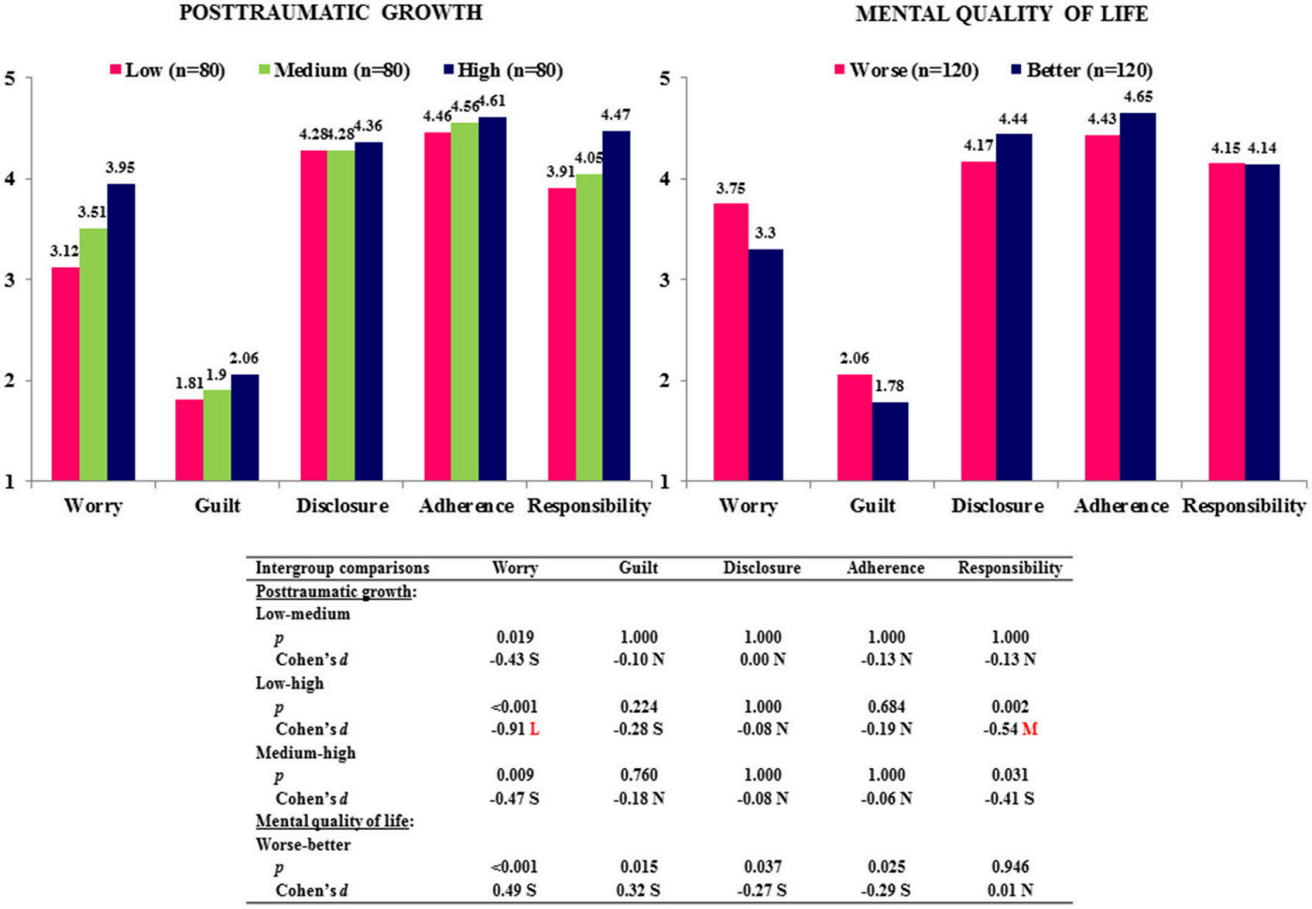
Influence of posttraumatic growth and mental quality of life on the TxEQ-Spanish subscales. N, Null effect size; S, Small effect size; M, Medium effect size; L, Large effect size.

As shown in Table 3, Figure 3, the posttraumatic growth main effect was significant for the dimensions worry (*p* < 0.001) and responsibility (*p* = 0.002). Scores on both dimensions were significantly higher in patients with high compared to low posttraumatic growth (worry, *p* < 0.001, *d* = −0.91, large effect size; responsibility, *p* = 0.002, *d* = −0.54, medium effect size). Posttraumatic growth was also positively correlated with worry (*r* = 0.37, *p* < 0.001), guilt (*r* = 0.13, *p* = 0.046), and responsibility (*r* = 0.25, *p* < 0.001) (Table 2).

The main effect of mental quality of life was significant regarding the dimensions worry (*p* < 0.001, *d* = 0.49, small effect size), guilt (*p* = 0.015, *d* = 0.32, small effect size), disclosure (*p* = 0.037, *d* = −0.27, small effect size), and adherence (*p* = 0.025, *d* = −0.29, small effect size) (Table 3, Figure 3). Patients with better mental quality of life scored higher on disclosure and adherence, and lower on worry and guilt, which corresponded to significantly positive or negative correlations between mental quality of life and above mentioned dimensions (Table 2).

### Predictors of posttraumatic growth

The results of the multiple linear regression analysis with posttraumatic growth as dependent variable and mental and physical quality of life, worry, guilt, disclosure, adherence, and responsibility as predictors are presented in Table 4. The final model [*F*_(3, 236)_ = 16.74, *p* < 0.001] consisted of the three significant predictors worry (*p* < 0.001), PCS (*p* = 0.017) and responsibility (*p* = 0.034). This model explained 17.5% (*R*^2^ = 0.175) of the variance observed in posttraumatic growth.

**Table 4:** Repercussions of liver transplantation (worry, guilt, disclosure, adherence, responsibility, MCS, and PCS) as predictors of posttraumatic growth.

**Predictor variables**	***B***	***SE***	**β**	***t*(*p*)**	***R^2^***	**Δ*R^2^***
Step 1					0.137	0.137
Worry	8.54	1.39	0.37	6.14 (<0.001)		
Step 2					0.160	0.023
Worry	9.24	1.40	0.40	6.58 (<0.001)		
PCS	0.32	0.13	0.15	2.54 (0.012)		
Step 3					0.175	0.016
Worry	8.17	1.48	0.35	5.51 (<0.001)		
PCS^a^	0.30	0.13	0.14	2.40 (0.017)		
Responsibility	2.92	1.36	0.13	2.14 (0.034)		

a *PCS, physical component summary*.

## Discussion

### Spanish adaptation and validation of the transplant effects questionnaire (TxEQ-spanish)

To date there is no standardized instrument available in Spain to assess the impact of transplantation on psychological well-being. Against this backdrop the current study aimed at translating the English original version of the TxEQ into Spanish and validate the Spanish version in a large sample of liver transplant recipients. In our methodological approach we followed the guidelines for the process of cross-cultural adaptation of self-report measures by Beaton et al. [24]. Thus, the adaptation procedure embraced five carefully designed steps, to ensure soundness of the final version. By means of this approach we ensured semantic, idiomatic experiential, and conceptual equivalence of both questionnaires and the comparability of responses in English and Spanish speaking populations. The verification of scaling requirements by analyzing the factor structure and reliablity of the TxEQ was implemented in the next step [24] to ensure psychometric quality of the Spanish version. CFA of the TxEQ-Spanish revealed adequate fit with the original English version of the TxEQ [1] and confirmed a robust five-factor structure. After careful analysis of the internal consistency of all subscales one item (item 8: “I do not have any feelings of guilt toward the donor”) showed a particularly low loading on the factor guilt and had to be excluded from the questionnaire. One possible reason for the low association of this item with the factor guilt might be that the translation into Spanish led to a double negative. This grammatical construction may have led to difficulties in comprehensibility, particularly in recipients with a low level of formal education, which made up 61.7% of patients in our sample. This modification of the Spanish version does not necessarily result in a relevant reduction in information provided by the subscale “guilt regarding the donor,” since it contains another item (item 13: “I feel guilty about having taken advantage of the donor”) with similar content. In the final version of the TxEQ-Spanish the internal consistency scores were satisfactory ranging from 0.77 to 0.91. Consistency scores were even higher than in the English original version [1] and the translated versions in German [2], Dutch [3], or Polish [4]. Therefore, our hypothesis that the TxEQ-Spanish has a factor structure (worry, guilt, disclosure, adherence, and responsibility) similar to the original version and shows satisfactory reliability has been confirmed.

### Mental quality of life, posttraumatic growth, and TxEQ-Spanish

Moreover, we analyzed the relationship between recipients' emotional reactions to transplantation, their mental quality of life and posttraumatic growth. In this context adherence to treatment and medication is of specific interest to optimize long-term outcome of transplantation. Analysis of variance showed an interaction effect between posttraumatic growth and mental quality of life on therapeutic adherence. Better mental quality of life was associated with more adherence merely in patients with medium posttraumatic growth. A positive association between mental health and adherence is in line with other studies [2, 6, 37–39] and confirms our hypothesis. However, it is not easy to explain, why this difference can only be seen in recipients with medium posttraumatic growth. One might argue that—taking mental health into account—there is no linear association between adherence and posttraumatic growth as Figure 3 might suggest, but rather a u-shaped or reverse u-shaped connection as shown in Figure 2. This relationship could be explained by the fact that adherence to medication worsens if the patient feels cognitively overloaded [40] and improves if simplification of medication regimen lowers the cognitive load [41]. Posttraumatic growth is defined by Tedeschi and Calhoun [10, 11] as a process, which takes time and absorbs a lot of cognitive resources to be able to form cognitive schemata reconciling existential opposites. If recipients are involved in posttraumatic growth, effects may largely depend on the extent of involvement. A moderate involvement in the process of posttraumatic growth (medium PTG) may be associated with disadvantages concerning treatment in recipients with worse mental health compared to those with better mental health, as worse mental health is associated with fewer mental resources and recipients may be overwhelmed by inner conflicts (“tunnel vision”). The mental overload could be mirrored in a positive response to items from the adherence scale such as “Sometimes I forget to take my anti-rejection medicines” and “When I am too busy I may forget my anti-rejection medicines.” Obviously, this explanation is highly speculative, and does not explain convincingly the lack of differences in the group with high PTG. Nevertheless, the aspect that posttraumatic growth is a resource consuming cognitive process needs to be taken into account to be able to understand the complexity of its effects. Significant predictors of PTG were the degree of physical complaints (PCS), worry, and responsibility with worry being the most important predictor. Posttraumatic growth implies a gain in self-awareness and spirituality, a re-definition of personal relationships and a greater appreciation of life with all its possibilities [10, 11]. At first sight it may be difficult to understand the relationship between worry and PTG. However, worries about the transplantation and all its implications, which are closely connected to the subjective experience of physical health (PCS), are the necessary predisposition for posttraumatic growth. The items “I am worried about damaging my transplant” or “I am hesitant to engage in certain activities because I am afraid of doing harm to my transplant” belonging to the worry-subscale demonstrate that worries affect all aspects of recipients' life. The process of posttraumatic growth is fueled by these repeated worries on the one hand and a sense of responsibility on the other, which enables the recipient to accept inner development as a personal task and not simply rely on fate. This responsibility also embraces a responsibility toward his own health and significant others. A sense of personal responsibility is closely connected to personality traits such as self-directedness and the psychological construct of self-efficacy, which both are strong predictors of psychological well-being and positive outcome after psychotherapy [42]. In summary, the prediction of posttraumatic growth by worry, physical health and responsibility gives further insight into those factors advancing positive change after liver transplantation [12, 14, 15, 19].

Regarding adherence our study confirmed a significant positive association with disclosure and a negative association with guilt [7, 43]. The difficulty of recipients to disclose their transplantation to others and a feeling of guilt toward the donor makes it difficult for recipients to exercise the adequate self-care. Thus, they may avoid taking their immunosuppressants in circumstances, where others may realize it or they may even ask themselves, whether they deserve to survive. Early identification of these problems and adequate treatment are crucial to avoid non-adherence resulting in rejection episodes and graft losses [44]. The importance of early psychological intervention can also be derived from the fact that a better mental quality of life is associated with more disclosure and less worry and guilt, which is in line with a previous study [2].

Our study shows several limitations. First, our sample consisted of liver transplant recipients, therefore our findings cannot be transferred to recipients of other organs. Second, all liver transplants were from deceased donors. Findings could be different in living donor liver transplantation [6, 7]. Third, recruitment of patients took place at a single site which may limit external validity of findings. Fourth, our cross-sectional study design does not allow for the investigation of longitudinal changes in respective questionnaires. Fifth, in our statistical analysis subgroups were created on the basis of sample distribution and not on the basis of validated cut-off values, which may restrict generalizability of study results.

Nevertheless our study successfully adapted and validated the TxEQ-Spanish in a large sample of liver transplant recipients, which allows for the future investigation of the psychological effects of transplantation by a psychometrically sound instrument in Spain.

## Author contributions

MÁP-S-G: Study concept and design, data analysis and interpretation, drafting of manuscript, manuscript revisions, drafting figures, final approval of version to be published. AM-R, MS-M, MB-M and MLA-N: Study concept and design, data analysis and interpretation, manuscript revisions, final approval of version to be published. MÁG-B: Institutional support, data collection, critical revision of article, final approval of version to be published. RC: Data analysis and interpretation, drafting of manuscript, critical revision of article, final approval of version to be published.

### Conflict of interest statement

The authors declare that the research was conducted in the absence of any commercial or financial relationships that could be construed as a potential conflict of interest.
