# Supplementary material for: Spanish Adaptation and Validation of the Transplant Effects Questionnaire (TxEQ-Spanish) in Liver Transplant Recipients and Its Relationship to Posttraumatic Growth and Quality of Life
Source: Front Psychiatry. 2018 Apr 18;9:148. doi: 10.3389/fpsyt.2018.00148 (PMC5915644; doi:10.3389/fpsyt.2018.00148)
Supplement: Supplementary file 1 [file DataSheet1.pdf]

Appendix 1. Cuestionario Efectos del Trasplante (TxEQ-Spanish).

Estamos interesados en conocer su ***punto de vista actual*** *de cómo* ***vive su experiencia con el trasplante de hígado****. Hay manifestaciones que otras personas han hecho sobre su experiencia con el trasplante. Por favor, indique en qué medida está o no de acuerdo con estas manifestaciones poniendo una* ***X*** *en el cuadro correspondiente. Gracias.*

	Totalmente de acuerdo	De acuerdo	Ni de acuerdo ni en desacuerdo	En desacuerdo	Totalmente en desacuerdo
1. En cuanto a mi trasplante, siento que llevo algo frágil dentro de mí.					
2. A veces creo que no necesito mi medicación anti-rechazo.					
3. Me da miedo practicar algunas actividades porque tengo miedo de dañar mi trasplante.					
4. Creo que tengo una responsabilidad hacia el equipo de trasplante de hacerlo bien.					
5. Estoy incómodo con que otras personas sepan que tengo un trasplante.					
6. Siento que debo algo al donante o a la familia del donante que nunca seré capaz de pagar.					
7. A veces olvido tomar mi medicación anti-rechazo.					
8. Estoy preocupado por dañar mi trasplante.					
9. Creo que tengo una responsabilidad hacia el donante o la familia del donante de hacerlo bien.					
10. Encuentro difícil adaptarme al tratamiento médico anti-rechazo prescrito.					
11. Vigilo mi cuerpo más de cerca que antes de ser trasplantado.					
12. Tengo dificultades en hablar sobre mi trasplante.					
13. Me siento culpable de haberme beneficiado del donante.					
14. Evito decirle a otras personas que tengo un trasplante.					
15. Me preocupo cada vez que mi médico cambia mi medicación anti-rechazo.					

	Totalmente de acuerdo	De acuerdo	Ni de acuerdo ni en desacuerdo	En desacuerdo	Totalmente en desacuerdo
16. El donante tuvo que sufrir para que yo me sienta mejor.					
17. Sigo preguntándome cuanto tiempo funcionará mi trasplante.					
18. A veces pienso que he "robado" una parte vital del donante.					
19. Cuando estoy demasiado ocupado puedo olvidar mi medicación anti-rechazo.					
20. Creo que tengo una responsabilidad hacia mis amigos y mi familia de hacerlo bien.					
21. A veces no tomo mi medicación anti-rechazo.					
22. Tengo el sentimiento de que el donante o la familia del donante tiene algún control sobre mí.					

TxEQ-Spanish Scoring

First stage:

Items	Score of items				
	Strongly agree	Agree	Uncertain	Disagree	Strongly disagree
1, 3, 4, 6, 8, 9, 11, 13, 15, 16, 17, 18, 20, 22	5	4	3	2	1
2, 5, 7, 10, 12, 14, 19, 21	1	2	3	4	5

Second stage: compute factors:

- Worry about transplant = Sum score of items 1, 3, 8, 11, 15, and 17 divided by 6. Higher scores show more worry.
- Guilt regarding the donor = Sum score of items 13, 16, 18, and 22 divided by 4. Higher scores show more guilt.
- Disclosure = Sum score of items 5, 12, and 14, divided by 3. Higher scores show more disclosure.
- Adherence = Sum score of items 2, 7, 10, 19, and 21, divided by 5. Higher scores show more adherence.
- Responsibility = Sum score of items 4, 6, 9, and 20, divided by 4. Higher scores show more responsibility.
