# Supplementary material for: Spanish Adaptation and Validation of the Transplant Effects Questionnaire (TxEQ-Spanish) in Liver Transplant Recipients and Its Relationship to Posttraumatic Growth and Quality of Life
Source: Front Psychiatry. 2018 Apr 18;9:148. doi: 10.3389/fpsyt.2018.00148 (PMC5915644; doi:10.3389/fpsyt.2018.00148)
Supplement: Supplementary file 2 [file DataSheet2.pdf]

Appendix 2. Comparison of TxEQ-Spanish and TxEQ-English on an item to item basis.

	Spanish version	English version
1.	En cuanto a mi trasplante, siento que llevo algo frágil dentro de mí	With regard to my transplant I feel that I am carrying around something fragile
2.	A veces creo que no necesito mi medicación anti-rechazo	Sometimes I think I do not need my anti-rejection medicines
3.	Me da miedo practicar algunas actividades porque tengo miedo de dañar mi trasplante	I am hesitant to engage in certain activities because I am afraid of doing harm to my transplant
4.	Creo que tengo una responsabilidad hacia el equipo de trasplante de hacerlo bien	I think that I have a responsibility to the transplant team to do well
5.	Estoy incómodo con que otras personas sepan que tengo un trasplante	I am uncomfortable with other people knowing that I have a transplant
6.	Siento que debo algo al donante o a la familia del donante que nunca seré capaz de pagar	I feel that I owe the donor/the donor's family something that I will never be able to repay
7.	A veces olvido tomar mi medicación anti-rechazo	Sometimes I forget to take my anti-rejection medicines
8.	Estoy preocupado por dañar mi trasplante	I am worried about damaging my transplant
9.	Creo que tengo una responsabilidad hacia el donante o la familia del donante de hacerlo bien	I think that I have a responsibility to the donor/the donors' family to do well
10.	Encuentro difícil adaptarme al tratamiento médico anti-rechazo prescrito	I find it difficult to adjust to taking my prescribed anti-rejection drug regime
11.	Vigilo mi cuerpo más de cerca que antes de ser trasplantado	I monitor my body more closely than before I had the transplant
12.	Tengo dificultades en hablar sobre mi trasplante	I have difficulty in talking about my transplant
13.	Me siento culpable de haberme beneficiado del donante	I feel guilty about having taken advantage of the donor
14.	Evito decirle a otras personas que tengo un trasplante	I avoid telling other people that I have a transplant
15.	Me preocupo cada vez que mi médico cambia mi medicación anti-rechazo	I worry each time my anti-rejection drug regime is altered by my doctor
16.	El donante tuvo que sufrir para que yo me sienta mejor	The donor had to suffer to make me feel better
17.	Sigo preguntándome cuanto tiempo funcionará mi trasplante	I keep wondering how long my transplant will work
18.	A veces pienso que he "robado" una parte vital del donante	Sometimes I think that I have 'robbed' the donor of a vital part
19.	Cuando estoy demasiado ocupado puedo olvidar mi medicación anti-rechazo	When I am too busy I may forget my anti-rejection medicines
20.	Creo que tengo una responsabilidad hacia mis amigos y mi familia de hacerlo bien	I think that I have a responsibility to my friends and my family to do well
21.	A veces no tomo mi medicación anti-rechazo	Sometimes I do not take my anti-rejection medicines
22.	Tengo el sentimiento de que el donante o la familia del donante tiene algún control sobre mí	I have the feeling that the donor/the donors' family has some control over me
